# Prognostic value of optical flow ratio for cardiovascular outcomes in patients after percutaneous coronary stent implantation

**DOI:** 10.3389/fcvm.2023.1247053

**Published:** 2023-12-01

**Authors:** Tianyu Hu, Qinghua Qiu, Nianjin Xie, Mingming Sun, Qianjun Jia, Meiping Huang

**Affiliations:** ^1^ Department of Catheterization Lab, Guangdong Cardiovascular Institute, Guangdong Provincial Key Laboratory of South China Structural Heart Disease, Guangdong Provincial People’s Hospital (Guangdong Academy of Medical Sciences), Southern Medical University, Guangzhou, China; ^2^ Department of Cardiology, Guangdong Cardiovascular Institute, Guangdong Provincial People’s Hospital (Guangdong Academy of Medical Sciences), Southern Medical University, Guangzhou, China

**Keywords:** coronary artery disease, optical coherence tomography, optical flow ratio, percutaneous coronary stent implantation, target vessel failure

## Abstract

**Background:**

The relationship between the optical flow ratio (OFR) and clinical outcomes in patients with coronary artery disease (CAD) after percutaneous coronary stent implantation (PCI) remains unknown.

**Objective:**

To examine the correlation between post-PCI OFR and clinical outcomes in patients with CAD following PCI.

**Methods:**

Patients who underwent optical coherence tomography (OCT) guided PCI at Guangdong Provincial People's Hospital were retrospectively and continuously enrolled. Clinical data, post-PCI OCT characteristics, and OFR measurements were collected and analyzed to identify predictors of target vessel failure (TVF) after PCI.

**Results:**

Among 354 enrolled patients, 26 suffered TVF during a median follow-up of 484 (IQR: 400–774) days. Post-PCI OFR was significantly lower in the TVF group than in the non-TVF group (0.89 vs. 0.93; *P* = 0.001). In multivariable Cox regression analysis, post-PCI OFR (HR per 0.1 increase: 0.60; 95% CI: 0.41–0.89; *P* = 0.011), large stent edge dissection (HR: 3.85; 95% CI: 1.51–9.84; *P* = 0.005) and thin-cap fibroatheroma (TCFA) (HR: 2.95; 95% CI: 1.19–7.35; *P* = 0.020) in the non-stented segment were independently associated with TVF. In addition, the inclusion of post-PCI OFR to baseline characteristics and post-PCI OCT findings improved the predictive power of the model to distinguish subsequent TVF after PCI (0.838 vs. 0.796; *P* = 0.028).

**Conclusion:**

The post-PCI OFR serves as an independent determinant of risk for TVF in individuals with CAD after PCI. The inclusion of post-PCI OFR assessments, alongside baseline characteristics and post-PCI OCT findings, substantially enhances the capacity to differentiate the subsequent manifestation of TVF in CAD patients following PCI.

## 1. Introduction

Percutaneous coronary stent implantation (PCI) is an important treatment for patients with coronary artery disease (CAD) (1). Not only the status of stent implantation, but also fractional flow reserve (FFR) following PCI, are related to future prognosis (2–4). However, traditional methods for assessing PCI results, such as coronary angiography and intravascular imaging, are primarily used to evaluate vascular structure and stent implantation status, but their ability to assess the physiological function of coronary is limited (5–7). With the emergence of functional tools based on angiography and endovascular imaging in recent years, this ability to assess the physiological function has been enhanced (8, 9).

In recent years, optical coherence tomography (OCT) has become increasingly popular as an intravascular imaging modality for microstructure assessment during PCI (3). Recent studies indicate that optical flow ratio (OFR), as an OCT-based method for fast computation of FFR, has a strong correlation with wire-based FFR (9, 10). Shunsuke et al. (11) suggest that OFR is an independent predictor of TVF in patients with acute coronary syndrome (ACS) after PCI. However, their study did not include non-ACS patients. Even though it is strongly advised to use OCT in ACS (12, 13), a subgroup of patients with chronic myocardial ischemia syndrome, including stable angina, also benefit from OCT (14, 15). However, the association between post-PCI OFR and CAD patients (including ACS and stable angina) remains unclear. Therefore, the objective of this study was to investigate the relationship between post-PCI OFR and clinical outcomes in CAD patients following OCT-guided PCI.

## 2. Methods

### 2.1. Study population

This was a retrospective and observational study aimed to investigate the association between post-PCI OFR and clinical outcomes in patients with CAD after OCT-guided PCI. Patients diagnosed with CAD who underwent OCT-guided PCI at Guangdong Provincial People's Hospital from January 2018 to July 2021 were consecutively enrolled. This study protocol complied with the Declaration of Helsinki and was approved by the Ethics Committee of Guangdong Provincial People's Hospital with the requirement for informed consent waived (KY-Q-2022-091-01).

#### 2.1.1. Inclusion criteria

Patients with CAD who underwent OCT-guided PCI and whose post-PCI OCT images were clear and analyzable. This clarity was attained through the successful removal of red blood cells from the OCT images, thereby enabling a precise visualization of the vascular tissue. In cases where a limited number of red blood cells remained, the accuracy of identifying the intimal tissue located behind them was still maintained.

#### 2.1.2. Exclusion criteria

Patients without stent implantation, those with non-post-PCI OCT images, or those with unanalyzable OCT images due to poor image quality were excluded. Patients with CAD due to in-stent restenosis were also excluded from the present study.

The target lesion was identified based on coronary angiography, electrocardiography, or coronary physiologic examination results. The indication for PCI was ultimately decided by the operator.

### 2.2. Analysis and definitions of angiographic view

The angiographic view that best exposed the severity of the stenosis was used for quantitative coronary angiography (QCA) analysis, using the QCA software (Beijing Sichuang Technology Imaging Systems, Beijing, China).

Diameter stenosis (DS) was determined as the quotient of the minimum diameter to the reference diameter. Multivessel disease was defined as DS exceeding 50% in at least two vessels.

### 2.3. Analysis and definitions of OCT images

Patients underwent OCT guided PCI. Following the completion of the PCI procedure, OCT images were obtained using an OCT system (OPTIS™; Abbott Vascular, Santa Clara, CA, USA) and Dragonfly™ Optis™ OCT imaging catheters. The OCT catheter was advanced at least 5–10 mm distal to the target stent, and blood in the lumen was emptied with automated contrast injection during image acquisition. Images were stored digitally offline and analyzed by two experienced investigators who were blinded to the angiographic data and clinical presentation.

**Figure 1:**
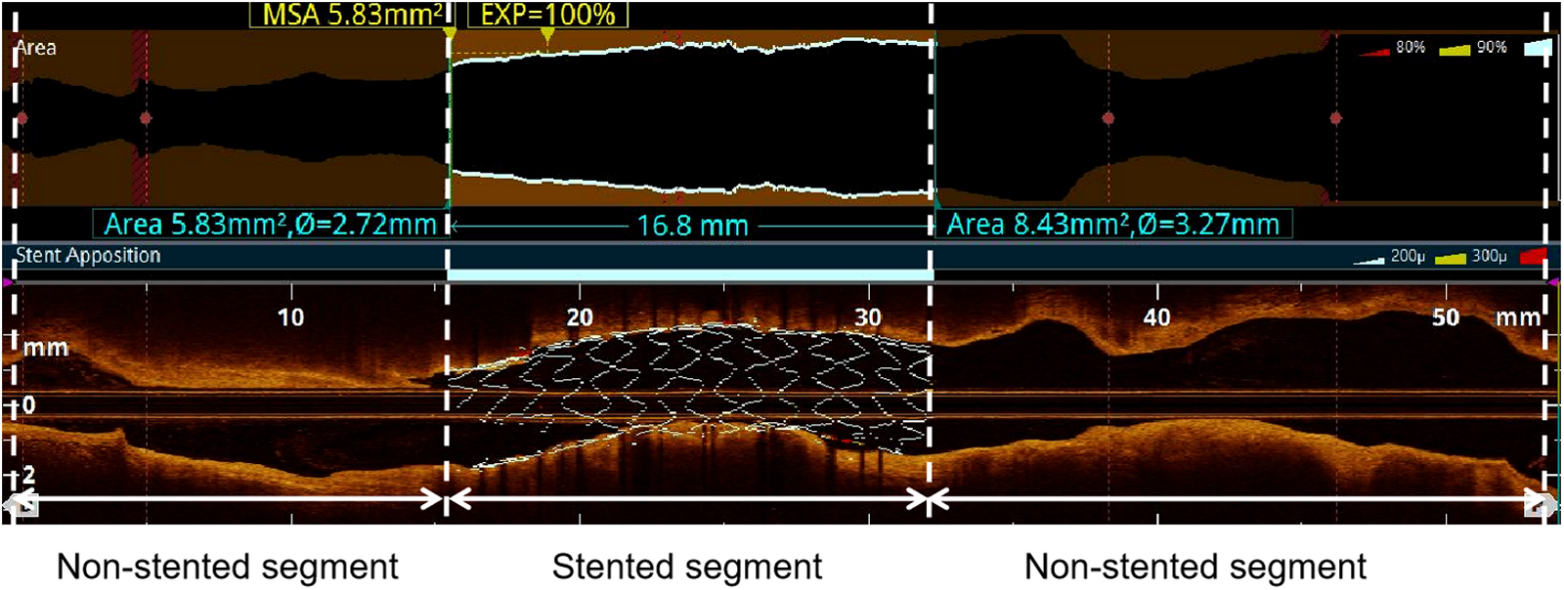
Subsegments analyzed by post-PCI OCT. The target vessel after PCI was divided into the following longitudinal subsegments: (1) stented segment; (2) non-stented segments. MSA: minimum stent area.

The target vessel was partitioned into two longitudinal subsegments after PCI: (1) the stented segment; (2) the non-stented segments (Figure 1). This study omitted the analysis of OCT results for branch vessels.

Stent expansion was quantified as the ratio of the minimum lumen area within the stent to the average reference lumen area (3, 16). The reference lumen was defined as the 5 mm lumen located distally and proximally from the stent edge. In-stent irregular protrusion was characterized as the presence of material with an uneven surface extending into the lumen between the stent struts (17, 18). To ensure accuracy, only protrusions within the stent with a maximum height of ≥200 μm were considered in the analysis, as some struts might be obscured within the intima. Malapposition was defined as the clear separation of struts from the vessel wall by ≥0.2 mm (17). Large stent edge dissection was characterized as the disruption of the luminal surface, exhibiting a visible flap (longitudinal extension >3 mm or extensive lateral >60° or deeper layers medial) at the stent edge (3). Thin-cap fibroatheroma (TCFA) was defined as plaque presenting a fibrous-cap thickness of <65 μm and a lipid arc of ≥180° (19, 20). In OCT imaging, we interpret areas of visually heightened signal intensity as potential indicators of macrophage infiltration. These regions present as distinct or continuous punctate patterns, where the signal intensity surpasses the speckle noise of the surrounding tissue. Such regions are commonly characterized as signal-rich (21).

### 2.4. OFR principle and plaque characterization analysis

**Figure 2:**
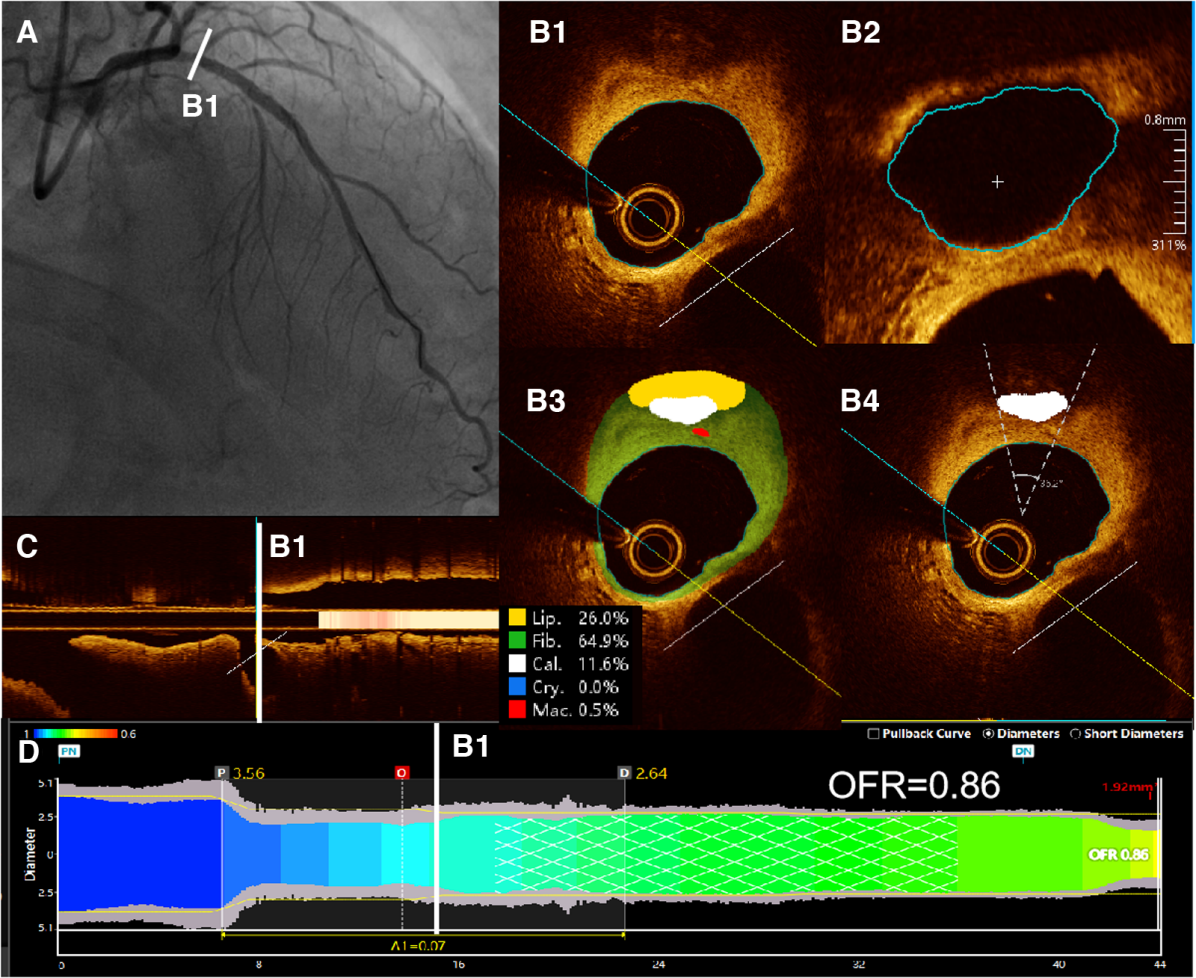
Examples of post-PCI OFR measurements and plaque composition analysis. (**A**) Coronary angiography showed residual stenosis in the proximal of LAD after PCI. (**B1**) All cross-sections from post-PCI OCT were automatically delineated for lumens and internal elastic lamina, and side branches were automatically detected. (**B2**) Then, the cut-plane perpendicular to the centreline of the side branch was reconstructed and the area of the side branch ostium in the cut-plane was computed. (**B3**) Plaque composition, including lipidic, fibrous, calcification, crystal and macrophage tissues, was detected and quantified by the software. (**B4**) Automatic computed values of cacification arc were shown on the corresponding cross sections. (**C**) Branches of the vessel were shown in the longitudinal view. (**D**) The computed post-PCI OFR measurements are color coded and displayed on the lumen-media diameter profiles. Post-PCI OFR of this example is 0.86. LAD, left anterior descending artery. OCT, optical coherence tomography. OFR, optical flow ratio.

In this study, the methodology for OFR computation and automatic plaque characterization, using OctPlus software (OctPlus1.0; Pulse Medical Imaging Technology, Shanghai, China), was previously described by Shengxian Tu et al. (22, 23) and Miao Chu et al. (24). All cross-sections from OCT were automatically delineated for lumens and internal elastic lamina, and side branches were automatically detected. Then, the area of the side branches ostium was calculated in the cut-plane perpendicular to the centreline of the side branches (23). In order to calculate the reference vessel size, the fractal laws of bifurcation were applied. The healthy lumen appears unaffected by stenosis, accounting for the inherent alteration in lumen dimensions caused by the step-down phenomenon during the traversal of bifurcations (22). In this model, the flow was assumed to be incompressible to follow the law of conservation of mass (25). The OFR value at each position along the vessel under investigation was calculated using a novel method derived from a validated computational FFR method. This involved applying a virtual volumetric flow rate at the inlet boundary. Specifically, the hyperemic volumetric flow rate was determined by multiplying the proximal reference lumen area obtained from OCT with a virtual hyperemic flow of 0.35 m/s (25). Following the computation, the reconstructed artery was color-coded based on the calculated OFR values (20). Plaque composition, including lipidic, fibrous, calcification, crystal and macrophage tissues, was subsequently detected and quantified by the software (20, 24). Examples of post-PCI OFR measurements and plaque composition analysis are presented in Figure 2. The aforementioned results were obtained by a proficient analyst who was blinded to the clinical outcomes.

To assess inter-observer and intra-observer consistency, we randomly selected 30 OCT images for analysis. Two experienced OCT imaging experts used OctPlus software to identify TCFA. Each researcher conducted a preliminary analysis at the initial time point and then repeated the analysis two weeks later.

### 2.5. Endpoint and follow-up

The primary endpoint of the study was target vessel failure (TVF), defined as the composite endpoint of cardiac death, target vessel-related myocardial infarction (TVMI), and ischemia-related target vessel revascularization (TVR) (11, 26). All deaths were considered cardiac deaths unless there was a clear non-cardiac cause. TVMI was identified by the presence of conclusive evidence of a target vessel subsequent to PCI. This evidence encompassed a notable elevation in creatine kinase-myocardial band (CK-MB) levels surpassing the upper reference limit, the observation of ST-segment elevation or depression on an electrocardiogram, and imaging results indicating fresh loss of viable myocardium or new regional wall motion abnormality. Procedural myocardial infarction (MI) refers to myocardial infarction that occurs as a result of a coronary intervention procedure, such as PCI, and was included in TVMI. TVR was defined as an unplanned repeat of PCI or bypass surgery of target vessels according to PCI. The event adjudication process was performed by an independent committee of experienced cardiologists who were blinded to the treatment assignments. They reviewed and analyzed all relevant clinical data, including medical records, laboratory results, and imaging studies, to determine whether each reported MI event met the predefined criteria for diagnosis. Any disagreements or uncertainties were resolved through discussion and consensus among the committee members. All patients underwent follow-up at least every year after PCI. Clinical outcomes were confirmed by medical record review, outpatient follow-up, and telephone visits.

### 2.6. Statistical analyses

The measurement data that adhered to a normal distribution were represented as mean ± SD, and the *t*-test was employed to compare groups. Continuous variables that did not adhere to a normal distribution were represented as median with an interquartile range (IQR) and analyzed using the Mann–Whitney *U*-test. Categorical variables were presented as frequencies, and group comparisons were conducted using the chi-square test or Fisher's exact test. All research variables were evaluated for their bivariate association with TVF. Those variables demonstrating a significant association at a level of *P* < 0.05 were included in the Cox regression model. Both univariate and multivariate Cox regression analyses were conducted to identify factors independently associated with TVF, and to calculate the corresponding hazard ratios (HR). Results were reported as HR with corresponding 95% confidence intervals (CI). Variables demonstrating a significant association in the univariate model (*P* < 0.05) were included in the multivariate model. The Cox regression model was applied using a backward elimination approach and was subjected to Bonferroni correction to adjust for multiple comparisons. Survival curves of TVF were generated through Kaplan-Meier analysis, and the log-rank test was utilized to compare TVF incidence disparities. The receiver operating characteristic (ROC) curve and the area under the ROC curve (AUC) were utilized to evaluate the added benefit of post-PCI OFR in conjunction with baseline characteristics and post-PCI OCT characteristics for the purpose of predicting TVF. The inter-observer and intra-observer variabilities for TCFA identification were evaluated using Cohen's Kappa statistic. The range of the Kappa value is between 0 and 1, where a value closer to 1 indicates better consistency.

In this research, the primary endpoint was assessed utilizing Cox regression analysis. Initially, we executed a univariate analysis to ascertain the correlation of each variable with TVF. Subsequently, variables that exhibited a significant correlation (*P* < 0.05) in the univariate Cox analysis, along with factors traditionally deemed clinically significant, were incorporated into the multivariate Cox regression model. This multivariate model employed a backward selection to identify which variables maintained a significant correlation with TVF upon controlling for other variables. Notably, even in the absence of statistical significance, factors traditionally considered clinically significant were included in the predictive model. The determination of statistical significance was undertaken via *P*-values. In both the univariate and multivariate Cox regression analyses, a *P*-value less than 0.05 was interpreted as statistically significant. Nevertheless, to adjust for the possibility of multiple comparisons, we implemented the Bonferroni correction.

## 3. Results

**Figure 3:**
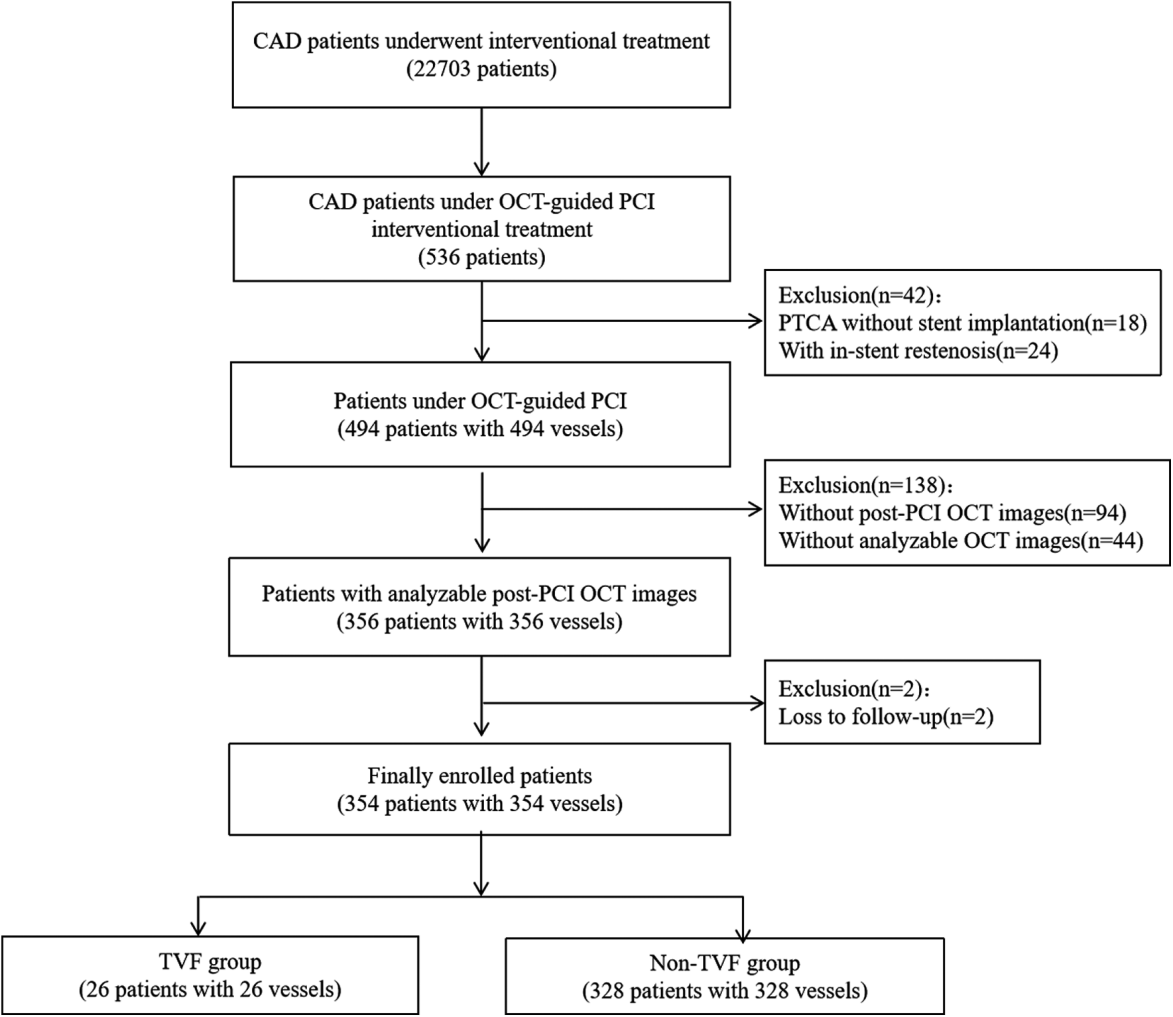
Patient flow diagram.

During the study period, a total of 494 consecutive patients underwent OCT-guided PCI. After exclusion of 140 patients, 354 patients with 354 vessels were finally included in the study (Figure 3). Table 1 provides a summary of the baseline, angiographic, and procedural characteristics. Table 2 presents a summary of the post-PCI OFR and OCT findings.

**Table 1:** Patient baseline, angiographic, and procedural characteristics.

	Overall (*n* = 354)	TVF (*n* = 26)	non-TVF (*n* = 328)	*P*-value
Male	294 (83.1)	21 (80.8)	273 (83.2)	0.786
Age, year	63 (55–71)	66 (61–73)	63 (55–71)	0.091
BMI, kg/m²	23.9 (21.8–25.8)	23.7 (20.5–25.3)	24 (21.9–25.9)	0.210
Comorbidity				
Hypertension	211 (59.6)	15 (57.7)	196 (59.8)	0.836
Diabetes mellitus	96 (27.1)	5 (19.2)	91 (27.7)	0.347
Smoking	117 (33.1)	5 (19.2)	112 (34.1)	0.120
Prior MI	31 (8.8)	2 (7.7)	29 (8.8)	0.842
Prior PCI	93 (26.3)	12 (46.2)	81 (24.7)	0.017
Prior CABG	1 (0.3)	0 (0)	1 (0.3)	1.000
Clinical presentation				
Stable angina	256 (72.3)	14 (53.8)	242 (73.8)	0.025
ACS	98 (26.7)	12 (46.2)	86 (26.2)	
ACS type				0.148
STEMI	42 (11.9)	6 (23.1)	36 (11.0)	
Non-STEMI	31 (8.8)	3 (11.5)	28 (8.5)	
Unstable angina	25 (7.1)	3 (11.5)	22 (6.7)	
Laboratory data				
LDL-C, mmol/L	2.7 (2.1–3.3)	2.3 (2.1–3.1)	2.7 (2.0–3.3)	0.485
Cholesterol, mmol/L	3.9 (3.4–5.1)	4.1 (3.5–5.1)	3.8 (3.4–4.6)	0.304
Creatinine, mg/dl	81.5 (70.0–95.0)	82.3 (73.1–101.7)	80.9 (69.4–95.0)	0.302
HGB, g/L	135 (121–145)	136.5 (111.8–147)	135 (121.5–144.5)	0.874
Pre-PCI CK-MB, U/L	12.0 (10.0–15.9)	14.5 (10.2–44.7)	12.0 (10.0–15.8)	0.075
LVEF, %	62 (57–67)	60.5 (49.5–63.3)	62 (58–67)	0.101
Lesion location				0.243
LAD	205 (58.1)	19 (73.1)	186 (56.9)	
LCX	35 (9.9)	1 (3.8)	34 (10.4)	
RCA	113 (32.0)	6 (23.1)	107 (32.7)	
QCA				
Lesion length, mm	26.7 (15.3–43.8)	30.8 (20.2–47.6)	26.4 (15.2–43.6)	0.371
Minimum diameter, mm	1.6 (1.2–2.1)	1.4 (1.1–1.6)	1.6 (1.2–2.1)	0.101
Reference diameter, mm	3.6 (3.3–4.0)	3.5 (3.0–3.7)	3.6 (3.3–4.1)	0.234
DS, %	57.1 (44.6–63.0)	56.5 (44.3–63.0)	60.0 (55.4–63.7)	0.220
Multivessel disease	223 (63.0)	18 (69.2)	205 (62.5)	0.494
Multiple stents	131 (37.0)	11 (42.3)	120 (36.6)	0.561
Stent length, mm	32 (23–42)	38 (28–46)	32 (22–41)	0.108
Pre-PCI TIMI flow				0.436
Grade 0 or 1	43 (12.1)	5 (19.2)	38 (11.6)	
Grade 2	11 (3.1)	0 (0)	11 (3.4)	
Grade 3	300 (84.7)	21 (80.8)	279 (85.1)	
Post-PCI TIMI flow				0.927
Grade 0 or 1	0 (0)	0 (0)	0 (0)	
Grade 2	1 (0.3)	0 (0)	1 (0.3)	
Grade 3	353 (99.7)	26 (100)	327 (99.7)	
Medication at discharge				
DAPT	352 (99.4)	25 (96.2)	327 (99.7)	0.337
Statin	343 (96.9)	24 (92.3)	319 (97.3)	0.416
Beta blocker	282 (79.7)	20 (76.9)	262 (79.9)	0.719

Values are expressed as median (IQR) or *n* (%).

ACS: acute coronary syndrome; BMI, body mass index; CABG, coronary artery bypass grafting; CK-MB, creatine kinase-myocardial band; DAPT, dual antiplatelet therapy; DS, diameter stenosis; HGB, haemoglobin; LAD, left anterior descending artery; LCX, left circumflex artery; LDL-C, low-density lipoprotein cholesterol; LVEF, left ventricular ejection fraction; MI, myocardial infarction; RCA, right coronary artery; STEMI, ST-segment elevation myocardial infarction; TVF, target vessel failure.

**Table 2:** Post-PCI OFR and OCT findings.

	Overall (*n* = 354)	TVF (*n* = 26)	non-TVF (*n* = 328)	95% CI	*P*-value
Target vessel					
Post-PCI OFR	0.92 (0.87–0.96)	0.89 (0.84–0.91)	0.93 (0.88–0.96)	−0.07, −0.02	0.001
Length of analyzable images, mm	53.4 (46.4–60.5)	53.8 (47.2–60.7)	53.1 (46.2–60.6)	−3.40, 2.60	0.615
Average lumen diameter, mm	3.1 (2.7–3.5)	2.9 (2.5–3.3)	3.1 (2.8–3.5)	−0.47, 0.34	0.074
Average lumen area, mm²	7.75 (6.21–9.35)	6.33 (5.33–8.42)	7.83 (6.29–9.37)	−2.39, 0.06	0.013
Proximal reference minimum lumen area, mm^2^	7.1 (5.3–9.5)	7.1 (5.4–9.2)	7.1 (5.3–9.6)	−2.19, 2.39	0.959
Distal reference minimum lumen area, mm^2^	4.6 (3.2–6.4)	3.9 (3.4–4.1)	4.7 (3.3–6.4)	−1.40, −0.63	0.089
MLA, mm²	5.3 (4.2–6.9)	4.0 (3.5–5.2)	5.5 (4.4–7.0)	−2.06, −0.57	0.001
Stented segment					
MSA, mm²	5.2 (4.3–6.6)	4.4 (3.7–5.6)	5.4 (4.4–6.6)	−1.69, −0.32	0.009
Mean stent area, mm²	8.2 (6.6–9.7)	7.8 (6.0–9.2)	8.2 (6.7–9.8)	−1.90, 0.34	0.128
Minimal expansion, %	58.0 (50.0–67.3)	58.5 (44.5–66.0)	58.0 (50.0–68.0)	−8.0, 4.0	0.415
Irregular protrusion	185 (52.3)	18 (69.2)	167 (50.9)	0.92, 5.13	0.072
Malapposition	211 (59.6)	16 (61.5)	195 (59.5)	0.48, 2.48	0.835
Non-stented segment					
Plaque detection					
Plaque volume, mm³	275.0 (221.5–329.4)	257.2 (201.6–302.3)	276.9 (223.7–334.0)	−52.35, 23.76	0.357
Fibrous tissue volume, mm³	186.7 (146.8–229.6)	177.9 (135.1–231.4)	188.1 (148.1–229.4)	−44.70, 15.19	0.452
Lipid plaque volume, mm³	47.6 (26.0–68.4)	40.6 (30.2–65.9)	48.1 (25.9–68.4)	−13.09, 8.96	0.944
Calcification volume, mm³	3.4 (1.2–8.6)	3.7 (1.6–6.6)	3.4 (1.1–8.9)	−1.36, 2.31	0.803
Crystal volume, mm³	0.04 (0.01–0.10)	0.05 (0.02–0.12)	0.03 (0.01–0.09)	−0.01, 0.08	0.363
Macrophage volume, mm³	0.23 (0.07–0.69)	0.24 (0.06–0.43)	0.23 (0.07–0.74)	−0.16, 0.14	0.315
Lipid					
Max area, mm²	4.3 (3.4–5.4)	3.8 (3.3–5.4)	4.4 (3.4–5.4)	−0.95, 0.09	0.287
Max volume, mm³	18.7 (8.2–33.5)	15.0 (10.4–26.8)	19.0 (8.1–33.8)	−9.03, 3.60	0.621
Max angle, °	223.2 (166.9–273.4)	233.3 (193.1–286.6)	221.2 (165.4–271.5)	−25.10, 46.30	0.360
Calcification					
Max area, mm²	1.3 (0.8–2.1)	1.4 (0.9–2.4)	1.3 (0.8–2.0)	−0.27, 0.61	0.400
Max volume, mm³	0.9 (0.3–2.6)	0.9 (0.3–2.4)	0.9 (0.3–2.7)	−0.42, 0.80	0.910
Max angle, °	86.5 (58.3–112.6)	93.5 (65.4–111.1)	85.5 (58.3–112.6)	−9.05, 18.10	0.552
Max thickness, mm	0.9 (0.7–1.1)	1.0 (0.8–1.2)	0.9 (0.7–1.1)	−0.05, 0.24	0.209
Qualitative findings					
TCFA	188 (53.1)	19 (73.1)	169 (51.5)	1.05, 6.24	0.034
Large stent edge dissection	35 (9.9)	7 (26.9)	28 (8.5)	1.53, 10.20	0.002

Values are median (IQR) or *n* (%).

CI, confidence interval; MLA, minimum lumen area; MSA, minimum stent area; Post-PCI OFR, percutaneous coronary stent implantation optical flow ratio; TCFA, thin-cap fibroatheroma; TVF, target vessel failure.

### 3.1. Outcomes

All enrolled patients completed the follow-up for at least 1 year. During a median follow-up of 484 (IQR: 400–774) days, TVF occurred in 26 (7.3%) patients. Specifically, the following were observed: cardiac death (4 patients), TVMI (12 patients), and TVR (10 patients).

### 3.2. Comparison of TVF and non-TVF groups

The incidence of previous PCI and ACS was significantly higher in the TVF group compared to the non-TVF group (Table 1).

Additionally, the TVF group exhibited significantly lower post-PCI OFR compared to the non-TVF group [0.89 (IQR: 0.84–0.91) vs. 0.93 (IQR: 0.88–0.96); *P* = 0.001] (Table 2).

These findings are presented in Table 2, which displays the results obtained from post-PCI OCT. In terms of the target vessel, the TVF group demonstrated significantly smaller average lumen area (*P* = 0.013), minimum lumen area (MLA) (*P* = 0.001), and minimum stent area (MSA) (*P* = 0.009) compared to the non-TVF group. Furthermore, the TVF group exhibited a higher prevalence of thin-cap fibroatheroma (TCFA) (*P* = 0.034) and large stent edge dissection (*P* = 0.002) compared to the non-TVF group.

### 3.3. Factors correlated with TVF

The findings of the univariable and multivariable Cox regression analyses pertaining to TVF are concisely presented in Table 3. The multivariable model demonstrated that previous PCI, ACS, post-PCI OFR, large stent edge dissection, and TCFA in the non-stented segment exhibited independent associations with TVF (Table 3).

**Table 3:** Cox regression analyses for factors associated with TVF after PCI in patients.

	Univariable regression			Multivariable regression		
	HR	95% CI	*P*-value	HR	95% CI	*P*-value
Traditional cardiovascular risk factors						
Male	1.17	0.44–3.11	0.748			
Age, year	1.04	1.00–1.08	0.053			
BMI, kg/m²	0.94	0.82–1.06	0.307			
Hypertension	1.08	0.49–2.34	0.852			
Diabetes mellitus	0.64	0.24–1.70	0.371			
Smoking	0.47	0.18–1.25	0.131			
Prior MI	0.82	0.19–3.46	0.785			
Prior PCI	2.51	1.16–5.43	0.019	3.85	1.70–8.73	0.001
ACS	2.19	1.01–4.77	0.048	2.60	1.23–5.96	0.018
Target vessel						
Post-PCI OFR(per 0.1 increase)	0.54	0.38–0.79	0.001	0.60	0.41–0.89	0.011
Average lumen area, mm²	0.78	0.64–0.96	0.018			
MLA, mm²	0.67	0.52–0.86	0.002			
Stented segment						
MSA, mm²	0.72	0.55–0.93	0.011			
Qualitative findings						
TCFA	2.48	1.04–5.90	0.040	2.95	1.19–7.35	0.020
Large stent edge dissection	3.91	1.64–9.32	0.002	3.85	1.51–9.84	0.005

ACS, acute coronary syndrome; CI, confidence interval; HR, hazard ratio; MLA, minimum lumen area; MSA, minimum stent area; Post-PCI OFR, percutaneous coronary stent implantation optical flow ratio; TCFA, thin-cap fibroatheroma.

**Figure 4:**
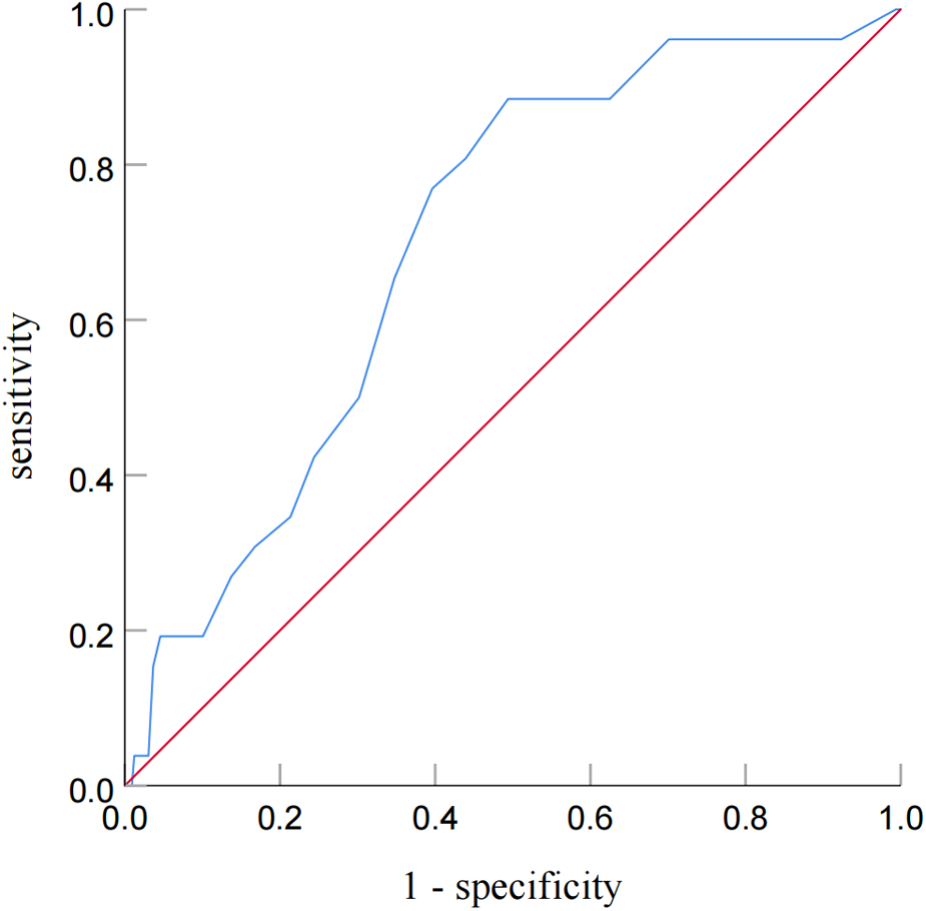
Receiver operating characteristic curve analysis for identifying patients with subsequent TVF from post-PCI OFR. Receiver-operating characteristic curve analysis showed that the cutoff value of the post-PCI OFR for identifying patients with subsequent TVF was 0.92.

ROC analysis revealed that the cutoff value of post-PCI OFR for the identification of patients with subsequent TVF was determined to be 0.92 (sensitivity: 60.4%; specificity: 76.9%; area under the curve: 0.701; *P* = 0.035) (Figure 4).

**Figure 5:**
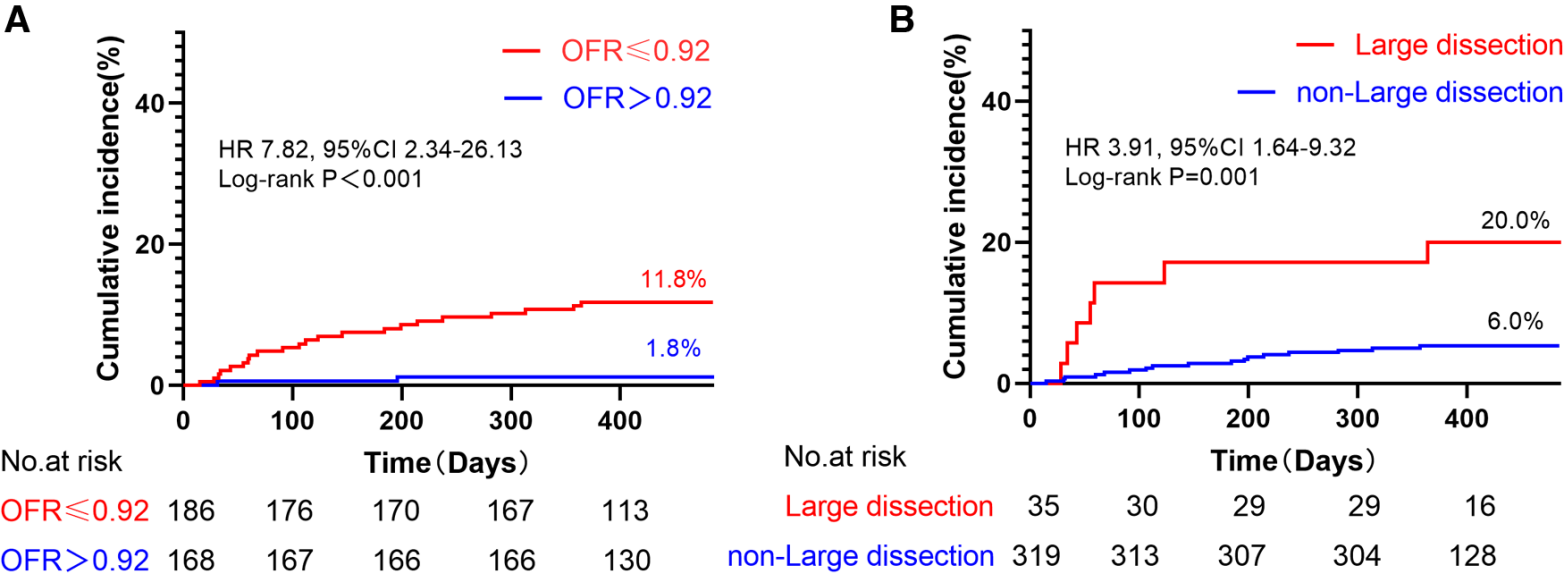
Kaplan–meier curves of TVF according to post-PCI OFR and large stent edge dissection. (**A**) The incidence of TVF was 6.6 times higher in vessels with low post-PCI OFR (≤0.92) than in those with high post-PCI OFR (>0.92). (**B**) The incidence of TVF 3.3 times higher in vessels with large stent edge dissection than in those without large stent edge dissection. It is noteworthy that these results were not adjusted for HRs.

Notably, the incidence of TVF was observed to be 6.6 times higher in vessels with low post-PCI OFR (≤0.92) compared to those with high post-PCI OFR (>0.92) (log-rank *P* < 0.001) (Figure 5A). Furthermore, it was observed that the occurrence of TVF was 3.3 times greater in vessels exhibiting large stent edge dissection compared to vessels without such dissection (log-rank *P* = 0.001) (Figure 5B).

### 3.4. The incremental value of OFR in identifying patients with TVF after PCI

**Figure 6:**
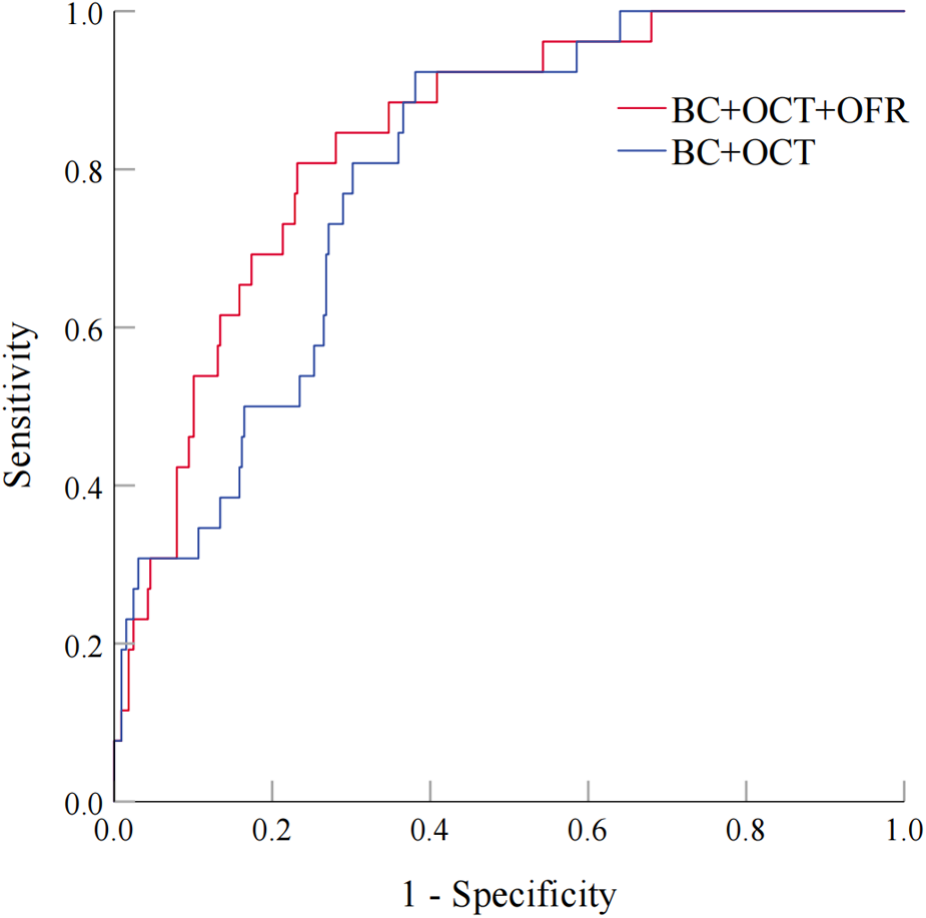
Evaluation of Two models for predicting TVF in CAD patients after PCI. Figure 6 compares two models. Model 1 uses baseline characteristics and post-PCI OCT findings, while Model 2 adds post-PCI OFR to Model 1. The comparison shows that Model 2 is better at predicting TVF in CAD patients after PCI (0.838 vs 0.796; *P* = 0.028), indicating that combining OFR with OCT and baseline characteristics improves prediction. BC, baseline characteristics; OCT, optical coherence tomography; OFR: optical flow ratio. Abbreviations as in Figure 6.

As shown in Figure 6, two models were compared. Model 1 was based on baseline characteristics and OCT results (large stent edge dissection and TCFA) following PCI, while Model 2 incorporated OFR after PCI into Model 1. The baseline characteristics included traditional cardiovascular risk factors (age, male, BMI, hypertension, diabetes mellitus, smoking, and prior MI), as well as baseline variables with statistical differences (prior PCI and ACS). The results demonstrated that Model 2 had superior ability in distinguishing subsequent TVF in CAD patients after PCI than Model 1 (0.838 vs. 0.796; *P* = 0.028). This indicated that the combination of OFR after PCI with OCT results and baseline characteristics significantly improved the ability to distinguish subsequent TVF in CAD patients.

### 3.5. Comparison of post-PCI OFR and plaque characteristics between patients with stable angina and ACS

In Table 4, we compared the post-PCI OFR and plaque detection results of patients with stable angina and ACS. There was no statistically significant difference in post-PCI OFR between patients with stable angina and ACS (*P* = 0.316). In terms of plaque detection, there was a significant difference in lipid plaque volume between the two groups of patients (*P* = 0.047), with a higher median lipid plaque volume in ACS patients. In addition, there was a significant difference in crystal volume between the two groups of patients (*P* = 0.001), with a higher median crystal volume in ACS patients. No significant differences were observed in other plaque detection parameters between the two groups.

**Table 4:** Post-PCI OFR and plaque detection between stable angina and ACS patients.

	Overall (*n* = 354)	Stable angina (*n* = 256)	ACS (*n* = 98)	*P*-value
Target vessel				
Post-PCI OFR	0.92 (0.87–0.96)	0.92 (0.87–0.96)	0.92 (0.87–0.96)	0.316
Plaque detection				
Plaque volume, mm³	275.0 (221.5–329.4)	271.2 (218.7–323.8)	282.1 (224.6–337.6)	0.255
Fibrous tissue volume, mm³	186.7 (146.8–229.6)	187.6 (148.1–229.4)	184.7 (138.2–230.4)	0.856
Lipid plaque volume, mm³	47.6 (26.0–68.4)	47.3 (25.4–64.7)	53.6 (28.2–86.4)	0.047
Calcification volume, mm³	3.4 (1.2–8.6)	3.5 (1.2–8.2)	3.1 (1.1–9.3)	0.636
Crystal volume, mm³	0.04 (0.01–0.10)	0.03 (0.01–0.09)	0.05 (0.02–0.16)	0.001
Macrophage volume, mm³	0.23 (0.07–0.69)	0.21 (0.06–0.68)	0.32 (0.07–0.83)	0.354
Lipid				
Max area, mm²	4.3 (3.4–5.4)	4.2 (3.3–5.2)	4.6 (3.5–5.8)	0.050
Max volume, mm³	18.7 (8.2–33.5)	18.1 (8.2–31.4)	20.4 (8.1–41.0)	0.226
Max angle, °	223.2 (166.9–273.4)	220.2 (165.4–271.0)	228.3 (178.5–228.3)	0.204
Calcification				
Max area, mm²	1.3 (0.8–2.1)	1.3 (0.8–2.0)	1.3 (0.8–2.2)	0.771
Max volume, mm³	0.9 (0.3–2.6)	0.9 (0.3–2.8)	0.8 (0.2–2.0)	0.228
Max angle, °	86.5 (58.3–112.6)	81.5 (50.8–107.1)	88.5 (62.3–112.6)	0.113
Max thickness, mm	0.9 (0.7–1.1)	0.9 (0.7–1.1)	0.9 (0.7–1.2)	0.954
Qualitative findings				
TCFA	188 (53.1)	131 (51.2)	57 (58.2)	0.238

ACS, acute coronary syndrome; Post-PCI OFR, percutaneous coronary stent implantation optical flow ratio; TCFA, thin-cap fibroatheroma.

### 3.6. Results of inter-observer and intra-observer consistency analysis

A high level of agreement was demonstrated between observers in the identification of TCFA. Two weeks prior, the Kappa value for inter-observer consistency was 0.862 (*P* < 0.001), and after two weeks, this consistency increased to 0.930 (*P* < 0.001). Over a two-week interval, the intra-observer consistency for the two observers was 1.0 (*P* < 0.001) and 0.930 (*P* < 0.001), respectively.

## 4. Discussion

The results of this study mainly show that: (1) In an unadjusted comparison among CAD patients who underwent PCI, the TVF group exhibited a significantly lower post-PCI OFR than the non-TVF group; (2) Upon adjustment for potential confounders through Cox regression analysis, it was found that post-PCI OFR is an independent predictor of TVF in CAD patients post-PCI; (3) The cut-off value of post-PCI OFR was 0.92, and the incidence of TVF in vessels with low post-PCI OFR was 6.6 times higher than that in vessels with high post-PCI OFR. (4) Post-PCI OFR demonstrated the incremental value in identifying patients with subsequent TVF beyond baseline characteristics and morphological OCT findings. (5) Despite similar post-PCI OFR in patients with stable angina and ACS, significant differences were observed in plaque characteristics, with ACS patients showing higher lipid plaque volume and cholesterol crystal volume. As far as we know, this is the first study to report the potential clinical predictive value of post-PCI OFR for subsequent TVF in CAD patients who underwent OCT-guided PCI.

Presently, intravascular imaging investigations have elucidated the intricate mechanisms underlying adverse outcomes in PCI within stented segments, and substantial enhancements in clinical outcomes have been evidenced through the use of intravascular imaging in comparison to solely relying on angiographic guidance (27). In contrast to PCI guided solely by angiography, intracavitary image-guided PCI has demonstrated noteworthy advancements in clinical outcomes (26, 28). Nevertheless, as PCI is a localized treatment, intravascular imaging typically concentrates on the target lesion or stented segment without considering the entirety of the target vessel (1, 9). In contrast, post-PCI FFR serves as an indicator of the remaining resistance within the entire vessel after maximal microvascular vasodilation (29). This measurement not only evaluates the level of stent optimization but also considers the presence of lesions in non-stent segments, both of which can impact future prognosis (30). The OFR method, which exclusively relies on quantitative lumen data derived from conventional OCT images for the computation of virtual FFR, offers unique advantages. Importantly, OFR is not just a numerical value. Along the direction of the vascular lumen, the color changes on the OFR correspond to changes in pressure ratio. Sudden color changes indicate sudden pressure ratio changes, pointing out the vascular segments that require our attention and may need further intervention to improve the final OFR. Unlike physiological measurements and OCT alone, it effectively mitigates superfluous congestion, providing a more comprehensive assessment of ischemia in patients with CAD subsequent to PCI (25, 31, 32).

We found that post-PCI OFR was a powerful independent factor associated with TVF. The possible reason is that stent underexpansion and residual lesions are significantly correlated with pressure drop, both of which may result in low physiological function value and lead to poor prognosis (9, 33). Therefore, in conjunction with luminal imaging-guided stent optimization, post-PCI OFR can provide insight into the potential benefit of additional PCI strategies in improving clinical outcomes (34, 35). Previous research has indicated a direct correlation between low post-PCI FFR and future adverse cardiac events (34). The specific cutoff value for post-PCI FFR may vary depending on factors such as the target lesion, vessel, and characteristics of the patients included in each study. Multiple investigations have demonstrated that the cutoff value for post-PCI FFR ranges from 0.89 to 0.91 for patients with coronary artery disease, encompassing both acute coronary syndrome and stable angina (2, 4, 33, 36). We found that the optimal cutoff value of post-PCI OFR was 0.92, which was slightly different from previous studies, and the reason might be related to the selection of patient population, number of cases and single center in this study.

Furthermore, this study demonstrated that incorporating post-PCI OFR into a model based on OCT findings and baseline characteristics (Model 2) significantly improved the ability to predict subsequent TVF in CAD patients compared to the model without post-PCI OFR (Model 1) (0.838 vs. 0.796; *P* = 0.028). These findings were consistent with the results of Shunsuke et al. (11).

In addition, this study shows that large stent edge dissection and TCFA in non-stented segment are independently associated with TVF in CAD patients after PCI. OCT can provide detailed information about high-risk plaques and accurately identify stent edge dissection (37, 38). In the TVF group, there is a high incidence of large stent edge dissections (26%) without further treatment. The possible reasons for this are that some surgeons believe that stent edge dissection does not require intervention with good flow on angiography, and previous studies support this view, which found no significant prognostic difference in stent edge dissection (38). But this study discovered that large stent edge dissection (length >3 mm or radian >60° or involving the media) is an independent related factor for TVF after PCI. The possible reason is that our study included large stent edge dissection as a research factor rather than with or without stent edge dissection. Large stent edge dissection is more likely to contribute to acute thrombotic events and affect blood flow, which is associated with poor prognosis (3, 27, 38). Our result is consistent with other studies reporting that severe stent edge dissection is associated with poor prognosis (38, 39). The result suggests that we need to pay more attention to large stent edge dissection, which may require further intervention.

Recent research findings indicate that TCFA serves as a strong predictor of plaque rupture, which is closely linked to significant adverse cardiovascular events (40). Furthermore, the outcomes of the current study demonstrate that TCFA in the non-stented segment is independently associated with TVF, aligning with previous reports (37, 41). The study cohort demonstrated a significant prevalence of TCFA, accounting for 53% of the entire cohort. However, in high-risk cohorts like the COMBINE OCT-FFR trial (41), only 25% of patients exhibited TCFA. This discrepancy may be attributed to the implementation of automated plaque detection, which enhances detection sensitivity and reduces assessment subjectivity, as well as the heightened risk profile of CAD patients with stents.

Our study findings reveal that while the post-PCI OFR is similar in patients with stable angina and ACS, their plaque morphology shows significant differences. Particularly, ACS patients demonstrated higher values in lipid plaque volume and cholesterol crystal volume, which might suggest that plaques in ACS patients tend to have a higher content of lipids and cholesterol crystals. These findings have important implications for treatment strategies and prognosis. Firstly, plaques rich in lipids are more prone to rupture, potentially leading to acute thrombotic events, which is of significant importance for risk assessment and treatment choices in ACS patients (12). Secondly, plaques rich in cholesterol crystals may be associated with inflammation, making them more prone to instability, which may require more intense anti-inflammatory treatment and closer monitoring (42). These differences underscore the need to consider not only the disease type but also the plaque morphology when treating patients with coronary artery disease. For plaques rich in lipids and cholesterol crystals, more aggressive lipid-lowering therapy may be needed, such as the use of drugs that lower low-density lipoprotein cholesterol, like proprotein convertase subtilisin/kexin type 9 inhibitors, as well as drugs that enhance plaque stability (43, 44).

Limitations of this study: First, because this is a retrospective observational study at a single center, there is inevitably selective bias, the relevant conclusions should be taken with caution and need to be further confirmed by a prospective multicenter randomized controlled trial. Second, due to the limited of OCT catheter pullback distance, not all lesions were included, which may lead to a certain deviation of the results. Third, one of the limitations of this study is that it primarily focuses on the evaluation of the main coronary arteries, with relatively limited assessment of the branch vessels. Further improvement is needed in prospective studies to enhance the evaluation of branch vessels. Fifth, our study did not delineate the components of proximal and distal plaques Fourth, a notable limitation is the non-routine use of Troponin T (TnT), a more sensitive and specific marker for MI, compared to the primarily used CK-MB. separately. We acknowledge that the small sample size of our study could lead to an overfitting issue in variable analysis, potentially resulting in decreased statistical power. Despite this, we recognize the significance of this aspect and pledge to incorporate it as much as possible in our future research. Sixth, because of the small sample size of non-ACS patients and the fact that the relevant studies on FFR did not perform a classificcation study for such patients (2, 4, 33, 35), we did not perform a classified analysis in this study, and we will perform analyzes in the future.

## 5. Conclusion

The presence of post-PCI OFR serves as an independent determinant of risk for TVF in individuals with CAD after PCI. Incorporating post-PCI OFR measurements alongside baseline characteristics and post-PCI OCT outcomes substantially enhances the capacity to differentiate the subsequent manifestation of TVF in patients with CAD after PCI. However, given the relatively low number of events in our study, these results should be considered hypothesis-generating and need to be confirmed in larger studies.

## Data Availability

The original contributions presented in the study are included in the article/Supplementary Material, further inquiries can be directed to the corresponding authors.
